# Catching and monitoring clinical innovation through performance indicators. The case of the breast-conserving surgery indicator

**DOI:** 10.1186/s13104-017-2597-6

**Published:** 2017-07-17

**Authors:** Anna Maria Murante, Silvio Candelori, Paola Rucci, Sabina Nuti, Manuela Roncella, Matteo Ghilli, Andrea Mercatelli, Maria Pia Fantini

**Affiliations:** 1 Scuola Superiore Sant’Anna, Istituto di Management, Laboratorio Management e Sanità, Piazza Martiri della Libertà 33, 56127 Pisa, Italy; 2 Department of Biomedical and Neuromotor Sciences, Alma Mater Studiorum-University of Bologna, Via San Giacomo 12, 40126 Bologna, Italy; 3 Breast Cancer Surgical Unit, University Hospital Trust of Pisa, Via Roma 67, 56126 Pisa, Italy; 4 Hospital Administration, University Hospital Trust “Careggi”, Largo Brambilla 3, 50134 Florence, Italy

**Keywords:** Performance indicators, Breast cancer, Breast conserving surgery, Healthcare quality, Professional involvement

## Abstract

**Background:**

The evolution in the surgical and diagnostic procedures, the attention to women’s preferences, the case mix, and differences in professional practices may lead to a variability in the quality of breast cancer clinical pathway. To catch and manage this variability it is important to use valid measures. The aim of this paper is to examine the concurrent validity of the breast-conserving surgery (BCS) indicator and to provide evidence to guide the quality improvement process.

**Methods:**

The BCS indicator was calculated using hospital discharge records (HDRs) and was validated against surgical registry (SR) data in a random sample of 336 women undergoing breast cancer surgery in 2012 in two Tuscan teaching hospitals. The concurrent validity of BCS was examined by cross-tabulating patients using the ICD-9 CM codes for breast surgery obtained from the two data sources.

**Results:**

The analysis, carried out involving breast cancer professionals, highlighted that the large majority of interventions coded as “mastectomies” in HDRs are in fact reconstructing procedures, including nipple-sparing, skin-sparing and skin-reducing mastectomies in SR. These results led us to refine the old algorithm, that calculates the proportion of breast-conserving surgery over the total number of breast interventions, and reclassify breast cancer surgical procedures into three categories: conservative, reconstructive and traditional mastectomy. Based on this new classification algorithm, the percentages of (I) reconstructive interventions were 16% at Florence TH and 38.3% at Pisa TH; (II) breast-conserving interventions were respectively 72.8 and 52.1%; and (III) mastectomies 11.2 and 9.6%. After adjusting for age in a logistic regression model, the percentages of reconstructive interventions at Florence and Pisa were respectively 22 and 34% and those of breast-conserving interventions 63 and 53%.

**Conclusions:**

Our results indicate that breast cancer care indicators should be refined by distinguishing reconstructive procedures (nipple/skin-sparing surgery with implant or breast tissue expander insertion) from traditional mastectomy. The involvement of breast care professionals in the choice of indicators proved to be crucial to capture the up-to-date breast cancer surgical practice and inform the quality improvement process.

## Background

Breast cancer is the most common cancer in women worldwide, with an estimated number of 464,000 cases in Europe in 2012 [1]. Due to its burden and related impact on healthcare services, quality of care assessment in breast cancer has become a central issue for policy makers and clinicians, to ensure that patients receive the highest standards of care [2–4]. Therefore quality targets and accreditation requirements for hospitals and breast units have been identified [5–7].

The European Society of Breast Cancer Specialist (EUSOMA) has defined quality indicators, linked to specific targets, in order to routinely measure and monitor the capacity of the breast units to ensure high-quality clinical outcomes [5, 6]. One of these indicators is the “Proportion of women with invasive breast cancer with a size <3 cm who underwent breast-conserving surgery (BCS)” and the target suggested by EUSOMA for this indicator is 70% (optimum level 80%) [5].

Recent European studies based on registries linked with claims data or hospital discharge records (HDRs) showed proportions of conservative interventions below these targets and with stable or decreasing trends [2, 8–10]. A recent study from Catalunia based on HDRs showed a significant increase in the percentage of patients treated with breast-conserving surgery (from 67.9% in 2005 to 74.0% in 2011). Of note, in this study, a decreasing trend was observed in high-tech hospitals, contrary to that observed in hospitals of medium and low complexity [11]. The BCS indicator computed using data from the nationwide mammography screening in Denmark ranged from 69.7 to 86.5% [12].

A large variability in BCS was also observed in Italy. This indicator, based on HDRs, is included in the performance evaluation system developed by the Management and Health Laboratory of the Scuola Superiore Sant’Anna of Pisa, Tuscany [13]. The Italian Parliamentary Commission inquiring on the efficiency and effectiveness of the National Health System [14] and, successively, a network of ten Italian Regions used the BCS indicator to measure the quality of surgery in breast cancer care. Moreover, a normative resolution of Tuscany Region, one of the ten Regions in the network, introduced the BCS indicator to monitor the performance of the breast units [15]. To this purpose, using routine administrative data may have several advantages, as they are readily available and can be used on a systematic basis [16]. In Tuscany Region, where the regional governance uses performance indicators, including the BCS indicator, to measure quality of care and set targets to guide the quality improvement process, health professionals are systematically involved in selecting and refining performance indicators. Indeed indicators should be used to guide the care improvement process and should be able to change the clinicians and healthcare organizations behavior [17, 18].

In the case of breast cancer surgery, the Tuscan clinicians agreed to use the BCS indicator. Inter-hospital comparison revealed a large variability among providers (from 65.9 to 90.3%) and low values for the three Tuscan teaching hospitals, surprising professionals themselves. Indeed, the finding of slightly lower proportions of BCS in high-complexity hospitals, which usually have high caseloads, is apparently counterintuitive and in contrast with the literature reporting that higher volumes are positively associated with better practices and outcomes [19] and in particular with a better chance of receiving a conservative intervention [20–22].

A possible source of variability in the BCS indicator is variability in coding diagnoses and procedures in the hospital discharge records database. Moreover, the case mix may be a possible, obvious, explanation of the differences between hospitals. Last but not least, in the recent years many changes occurred in the breast cancer care management, in particular the combination of breast oncologic and reconstructive surgery has revolutionized the surgical approach to breast cancer.

The aim of this paper is to examine the concurrent validity of the BCS indicator to determine whether it reflects accurately the ongoing choices in surgical practice, in particular in the teaching hospitals.

This was done by analyzing the possible reasons of the low proportions of BCS in two high-volume teaching hospitals of Tuscany Region, as measured by the traditional BCS indicator, to determine whether the BCS indicator is up-to-date and valid to monitor appropriateness and quality of breast cancer care and to guide the quality improvement process.

## Methods

### Setting

The Italian National Health Care System (NHS) is a public health system providing universal coverage for comprehensive and essential health services through general taxation. This public system should ensure the achievement of equitable access to health care regardless of individual ability to pay or other characteristics such as income and region of residence. Since the early 1990s, a strong policy of decentralization has been taking place in Italy and powers have gradually shifted from the state to the 21 Italian regions. These regions now have political, administrative, and financial responsibility regarding the provision of health care.

Since 2008 a network of Italian Regions have been using a performance evaluation system, developed by Laboratorio Management e Sanità of the Scuola Superiore Sant’Anna in Pisa (Italy) [23] as a governance tool aimed to manage and improve the performance of healthcare organizations and ensure appropriate and equitable answers to population’ needs. Since 2012, these ten Regions have included the BCS indicator among the performance indicators. In particular, Tuscany Region has undertaken a process of engagement of the breast cancer care professionals by sharing indicators to measure quality of care. The engagement of professionals is essential to drive improvement strategies, changing clinicians’ behaviors and practices [24–28].

### Design and data sources

In the 2014 Tuscan cancer care professionals took part in a study promoted by the Regional Health Department with the aim to analyze the determinants of the inter-hospital variability of BCS indicator. Using a shared protocol, the professionals and a team of researchers extracted and analyzed anonymous data from both regional and local databases including information on diagnosis and surgical procedures delivered to women with breast cancer during the hospital stay. The data sources were:

1. The HDR database, which routinely collects data on hospital discharges, including referral source, discharge status, up to six discharge diagnoses (ICD-9-CM), up to six hospital procedures (ICD-9-CM) and patient’s demographic data.
2. The surgical registry (SR) database, which includes a description of the interventions, the date and the time of the interventions.
3. The pathology registry database includes the histological diagnosis, the presence of a sentinel lymph node and/or other lymph nodes, the tumor size and staging.

A random sample of 400 cases was extracted from the HDR database of the two Teaching Hospitals in Tuscany (Pisa TH and Florence TH). Women diagnosed with breast cancer (ICD-9-CM code 174*), who underwent a surgical intervention (ICD-9-CM procedure codes 85.2*, 85.3*, 85.4*) in the year 2012, were considered eligible for the study. We included only incident interventions, and excluded re-interventions within 4 months. The BCS indicator, based on the existing algorithm for HDR data, was first computed. It distinguishes conservative surgical interventions (ICD-9-CM procedure codes 85.2* and 85.3*) from traditional mastectomy (ICD 9-CM procedure code 85.4).

Data from the SR were then linked with the HDRs of Pisa and Florence samples by using as a key the medical record’s code. Then, using SR information, we coded interventions as conservative when quadrantectomy or lumpectomy was reported, reconstructive when nipple sparing, skin sparing mastectomy or skin-reducing mastectomy with prosthesis insertion were reported, and mastectomy elsewhere.

The validation of the BCS indicator was done by cross-tabulating patients using the HDR classification (breast-conserving and mastectomy procedures) and that derived from SR. All discrepant cases were examined in detail in order to identify different coding practices of interventions.

After a review of the textual description of the SR interventions, the old BCS indicator was refined by distinguishing reconstructive procedures from conservative procedures and mastectomies and the corresponding ICD-9-CM were used to define a new algorithm (Table 1). In this algorithm, based on HDR data, procedures were defined as conservative if they included excision or destruction of breast tissue or unilateral/bilateral reduction mammoplasty; reconstructive if they included implant breast reconstruction or the insertion of breast tissue expander, and mastectomies elsewhere. Lastly, after calculating the indicator using the new algorithm, we compared the probabilities of the different surgical procedures between the two hospitals after adjusting for age using logistic regression models. Data were analyzed using SAS Version 9.2.

**Table 1:** ICD-9CM codes for conservative, reconstructive and mastectomy interventions

Type of intervention	Old algorithm’s HDR codes	New algorithm’s HDR codes
Conservative	85.2* Excision or destruction of breast tissue or 85.3* Reduction mammoplasty and subcutaneous mammectomy	85.2* Excision or destruction of breast tissue or 85.31 Unilateral reduction mammoplasty or 85.32 Bilateral reduction mammoplasty or
Reconstructive		85.4* + 85.53 Unilateral breast implant or 85.4* + 85.54 Bilateral breast implant or 85.4* + 85.95 Insertion of breast tissue expander or 85.33 Unilateral subcutaneous mammectomy with synchronous implant or 85.35 Bilateral subcutaneous mammectomy with synchronous implant 85.34 Other unilateral subcutaneous mammectomy or + 85.53/85.54/85.95 85.36 Other bilateral subcutaneous mammectomy + 85.53/85.54/85.95
Mastectomy	85.4* Mastectomy	85.4* Mastectomy

## Results

In 2012, 622 interventions were performed at Florence teaching hospital (TH) and 611 at Pisa TH. After randomly extracting 200 records from the HDR database for each hospital and excluding re-interventions (n = 23 for Florence TH) and patients for whom SR information was not available (n = 8 for Florence TH and n = 33 for Pisa TH), the final samples were N = 169 at Florence TH and N = 167 at Pisa TH. Mean age ± SD of women was 60.1 ± 13.6 at Florence TH and 58.0 ± 13.3 for Pisa TH (*t* test = 1.46, p = 0.146). Tumor size was 1.67 ± 1.01 for patients discharged from Florence TH and 1.51 ± 0.81 for patients from Pisa TH (Wilcoxon rank-sum test = 21,773.5, p = 0.1786). Staging distribution was similar in the two hospitals, with no statistically significant difference (χ^2^ test = 6.7023, p = 0.082), as shown in Table 2.

**Table 2:** Tumor stage distribution for Florence TH and Pisa TH

Tumor stage	Teaching hospital	
	Florence	Pisa
0	2.74% (4)	9.43% (15)
I	54.79% (80)	54.72% (87)
II	31.51% (46)	28.30% (45)
III or more	10.96% (16)	7.55% (12)
Total	100% (146)	100% (159)

χ^2^ test = 6.7023, p = 0.082

The BCS indicator based on HDRs was 73.4% at Florence TH and 62.9% at Pisa TH (Table 3), and that based SR data was 73.4% at Florence TH and 52.1% at Pisa TH.

**Table 3:** Cross-tabulation of the number of mastectomies and conservative surgery as derived from the existing algorithm based on HDR data and from surgical registry data in the two study hospitals

	Surgical registry			Total
	Conservative	Reconstructive	Mastectomy	
Florence				
HDR database				
Conservative: (85.2*, 85.3*)	72.8% (123)	0.6% (1)	0	73.4% (124)
Mastectomy (85.4*)	0.6% (1)	17.8% (30)	8.3% (14)	26.6% (45)
Total	73.4% (124)	18.3% (31)	8.3% (14)	100% (169)
Pisa				
HDR database				
Conservative: (85.2*, 85.3*)	52.1% (87)	10.8% (18)	0	62.9% (105)
Mastectomy (85.4*)	0	27.5% (46)	9.6% (16)	37.1% (62)
Total	52.1% (87)	38.3% (64)	9.6% (16)	100% (167)

A large number of cases classified as “mastectomies” in HDRs (68.9% at Florence TH and the 74.2% at Pisa TH) proved to be reconstructive procedures (including nipple-sparing, skin-sparing and skin-reducing mastectomies) in the SR. Thus, after applying the new algorithm we reclassified HDR breast cancer surgical procedures into three categories: conservative, reconstructive and traditional mastectomy. Interventions with both conservative and reconstructive codes were recoded as reconstructive; those with both conservative and mastectomy codes were recoded as mastectomy and lastly interventions with both mastectomy codes and 85.33 or 85.35 were recoded as reconstructive.

Based on this new classification algorithm, the percentages of (I) reconstructive interventions were 16% at Florence TH and 38.3% at Pisa TH; (II) breast-conserving interventions were respectively 72.8 and 52.1%; and (III) traditional mastectomies 11.2 and 9.6% (Table 4). To be consistent with EUSOMA BCS indicator, we conducted a secondary analysis by excluding 12 patients in Florence TH and eight in Pisa TH with tumors >3 cm. The percentage of reconstructive interventions changed to 15.3% at Florence TH and 39% at Pisa TH; breast-conserving interventions were 75.8 and 52.8%; and traditional mastectomies 8.9 and 8.2%. Percentages calculated with the two methods did not differ significantly.

**Table 4:** Cross-tabulation of the number of mastectomies and conservative surgery as derived from HDR data and surgical registry data in the two study hospitals, using the new classification algorithm

	Surgical registry			Total
	Conservative	Reconstructive	Mastectomy	
Florence				
HDR database				
Conservative	72.8% (123)	0	0	72.8% (123)
Reconstructive	0	16% (27)	0	16% (27)
Mastectomy	0.6%^a^ (1)	2.4%^b^ (4)	8.3% (14)	11.2% (19)
Total	73.4% (124)	18.3% (31)	8.3% (14)	100% (169)
Pisa				
HDR database				
Conservative	52.1% (87)	0	0	52.1% (87)
Reconstructive	0	38.3% (64)	0	38.3% (64)
Mastectomy	0	0	9.6% (16)	9.6% (16)
Total	52.1% (87)	38.3% (64)	9.6% (16)	100% (167)

^a^This case was a quadrantectomy wrongly classified as 85.4*

^b^In 4 cases, in addition to code 85.4, we found codes 85.89 (other mammoplasty), 86.70 (free flap reconstruction) or 86.93 (insertion of generic tissue expander)

**Fig. 1:**
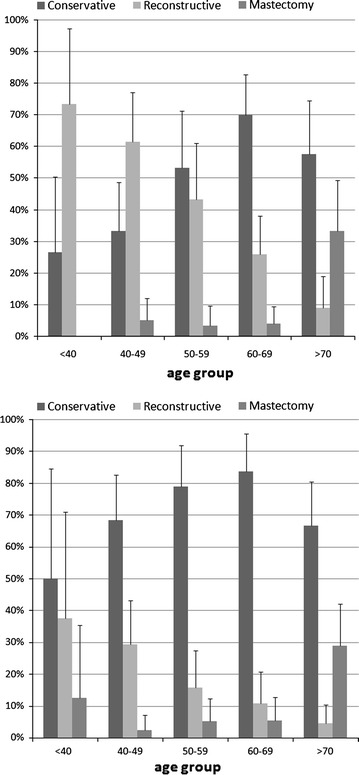
Percentage of interventions by age group in the teaching hospital of Florence (*top*) and Pisa (*bottom*)

In order to examine the breast surgery practice of the two hospitals in detail, the three indicators were calculated for five age groups. Results indicate that reconstructive interventions were more common among women under 40 years as compared with those in other age groups, at Florence TH and Pisa TH (Fig. 1).

On the contrary traditional mastectomies were more common among older patients, being performed in 29 and 33% of patients >70 years. After adjusting for age in a logistic regression model, the percentages of reconstructive interventions at Florence and Pisa were respectively 22 and 34% and those of breast-conserving interventions and 63 and 53%.

## Discussion

Breast-conserving surgery followed by adjuvant radiotherapy has been the gold standard for invasive and unifocal breast cancer since the 1980s. The indications of the Italian Ministry of Health, issued in 2010 (http://www.salute.gov.it/imgs/C_17_pubblicazioni_1700_allegato.pdf), read: ‘Surgery is based on partial resection or quadrantectomy when the size of the tumor is within the anatomical limits of a conservative surgery. The ratio between the breast volume and the size of the excision should be favorable to the complete removal of the neoplasm with an acceptable esthetic result.’

Thus, in our algorithm we labeled as conservative all local interventions including lumpectomy quadrantectomy or mammoplasty reduction.

However, in recent years, the preoperative genetic screening and diagnostic imaging, the development of new surgical techniques and the higher attention on the esthetic and psychological outcomes have produced the spread of reconstructive procedures [29, 30]. Therefore, updating quality of care indicators is necessary in order to make them more sensitive to clinical practice changes and technical progress.

Our results indicate that the apparently low percentage of conservative procedures in teaching hospital is due to the inclusion of reconstructive procedures among mastectomies.

The update of the coding algorithm allowed us to establish that traditional mastectomies were about 10% in both hospitals, in line with EUSOMA standards.

We argue that the *breast*-*conserving procedures* indicator should be complemented with the *reconstructive procedures* indicator to provide a more accurate description of the ongoing breast cancer surgery practice in the hospital and to monitor inter-hospital variability.

The present study shows how immediate breast reconstruction is an increasingly common practice in breast cancer surgery, performed in 16% (Florence TH) and 38.3% (Pisa TH) of women, with a peak at younger ages. Our results are consistent with Yang et al., who reported that the percentage of immediate breast reconstruction declines with age, and has a maximum in the age group <40 years [31].

We found that the large difference in the percentage of reconstructive procedures between the two study hospitals was only partially accounted by the lower mean age of women undergoing surgery at Pisa TH, suggesting that other clinical factors, organizational factors or patients’ decisions play an important role. One explanation is that the diagnostic procedures adopted in the two hospitals differed: MRI and preoperative genetic screening was more frequent in Pisa than in Florence, as reported by the breast unit teams of the two hospitals. The more frequent use of MRI facilitates the identification of multifocal tumors and might have led to the surgeon’s decision to undertake reconstructive surgery instead of a conservative procedure.

Our study has several strengths that should be considered. First, the Italian National Health System has a universal coverage; therefore, we could assume that individual economic resources did not account for the variability of the indicator among hospitals. Second, results are based on HDRs, a population-based database, that has been increasingly used to assess quality of care at national level and proved to have a good reliability for breast cancer cases [32]. The development and update of measures to monitor and assess the quality of healthcare pathways, such as cancer care, are crucial steps for the quality improvement and have to be shared with health professionals. Indeed, the improvement strategies are more effective when the professionals involved in the care pathway take part in the systematic data analysis by means a peer-review process. This approach makes health professionals more aware of the quality of services they contribute to produce and more likely to adopt or implement quality improvement strategies [28].

Concerning limitations, the use of HDRs has also shortcomings. Because of the administrative nature of this database, there could be some variability in ICD-9-CM coding practices between hospitals and professionals and miscoding can be possible. Moreover, administrative database does not include clinical information on tumor size or staging. Therefore, comparison between our indicators and EUSOMA indicators might be hindered by the difference in the denominator definition. In fact, when we calculated our indicators using the same criteria as EUSOMA to exclude tumors >3 cm, we found some discrepancies with those calculated using only HDR data, but differences were not significant. Lastly, an easier access to plastic surgery for patients admitted to teaching hospital could be a bias for breast surgery indicators when comparing teaching and non-teaching hospital. However working on the systematic review of coding procedures and disseminating the updated algorithms among professionals can reduce mistakes in HDR coding and, in turn, partial out some of the unwarranted variability in results.

Future studies are needed to examine in deeper detail the impact that clinical, organizational and preference related reasons have on the surgeon’s and women’s choice of the surgical procedure and to observe whether the monitoring of the three procedures over time will allow reducing the variability in the surgical practice across hospitals.

## Conclusions

The engagement of professionals in the revision process of the BCS indicator allowed to detect effectively the “bug” of the BCS indicator and to modify rapidly the algorithm used for its calculation. The update of the criteria for monitoring the breast cancer surgical practices allowed (I) catching clinical innovation, (II) ensuring that measurement and targets were set in a correct way, and (III) addressing the improvement process. Considering how strong the effect of control measures in guiding professionals’ behavior can be, it is important to avoid misleading indicators where maybe the targets are achieved but the real point is missed [33]. This is possible if professionals themselves consider these indicators as a work tool. Lastly, only the synergic work among professionals, managers and researchers can ensure the use of a common language that is the first step of a common path towards the improvement of quality of the health care services.

## Abbreviations

BCS: breast-conserving surgery
HDR: hospital discharge record
SR: surgical registry
NHS: National health care system
TH: teaching hospital

## References

[1] Ferlay J, Steliarova-Foucher E, Lortet-Tieulent J, Rosso S, Coebergh JWW, Comber H, Forman D, Bray F (2013). Cancer incidence and mortality patterns in Europe: estimates for 40 countries in 2012. Eur J Cancer.

[2] Stordeur S, Vrijens F, Devriese S, Beirens K, Van Eycken E, Vlayen J (2012). Developing and measuring a set of process and outcome indicators for breast cancer. Breast.

[3] Ferrua M, Couralet M, Nitenberg G, Morin S, Serin D, Minvielle E (2012). Development and feasibility of a set of quality indicators relative to the timeliness and organisation of care for new breast cancer patients undergoing surgery. BMC Health Serv Res.

[4] McCarthy M, Gonzalez-Izquierdo A, Sherlaw-Johnson C, Khachatryan A, Coleman MP, Rachet B (2008). Comparative indicators for cancer network management in England: availability, characteristics and presentation. BMC Health Serv Res.

[5] Del Turco MR, Ponti A, Bick U, Biganzoli L, Cserni G, Cutuli B, Decker T, Dietel M, Gentilini O, Kuehn T, Mano MP, Mantellini P, Marotti L, Poortmans P, Rank F, Roe H, Scaffidi E, van der Hage JA, Viale G, Wells C, Welnicka-Jaskiewicz M, Wengstöm Y, Cataliotti L (2010). Quality indicators in breast cancer care. Eur J Cancer.

[6] Wilson ARM, Marotti L, Bianchi S, Biganzoli L, Claassen S, Decker T, Frigerio A, Goldhirsch A, Gustafsson EG, Mansel RE, Orecchia R, Ponti A, Poortmans P, Regitnig P, Rosselli Del Turco M, Rutgers EJT, van Asperen C, Wells CA, Wengström Y, Cataliotti L (2013). The requirements of a specialist Breast Centre. Eur J Cancer.

[7] Blamey RW, Cataliotti L (2006). EUSOMA accreditation of breast units. Eur J Cancer.

[8] Van Hoeve J, de Munck L, Otter R, de Vries J, Siesling S (2014). Quality improvement by implementing an integrated oncological care pathway for breast cancer patients. Breast.

[9] Sacerdote C, Bordon R, Pitarella S, Mano MP, Baldi I, Casella D, Di Cuonzo D, Frigerio A, Milanesio L, Merletti F, Pagano E, Ricceri F, Rosso S, Segnan N, Tomatis M, Ciccone G, Vineis P, Ponti A (2013). Compliance with clinical practice guidelines for breast cancer treatment: a population-based study of quality-of-care indicators in Italy. BMC Health Serv Res.

[10] Jeevan R, Cromwell DA, Browne JP, Caddy CM, Pereira J, Sheppard C, Greenaway K, van der Meulen JHP (2014). Findings of a national comparative audit of mastectomy and breast reconstruction surgery in England. J Plast Reconstr Aesthet Surg.

[11] Escribà JM, Pareja L, Esteban L, Gálvez J, Melià A, Roca L, Clèries R, Sanz X, Bustins M, Pla MJ, Gil MJ, Borrás JM, Ribes J (2014). Trends in the surgical procedures of women with incident breast cancer in Catalonia, Spain, over a 7-year period (2005–2011). BMC Res Notes.

[12] Langagergaard V, Garne JP, Vejborg I, Schwartz W, Bak M, Lernevall A, Mogensen NB, Larsson H, Andersen B, Mikkelsen EM (2013). Existing data sources for clinical epidemiology: the Danish quality database of mammography screening. Clin Epidemiol.

[13] Nuti S, Bonini A. Il Sistema Di Valutazione Della Performance Dei Sistemi Sanitari Regionali : Basilicata, Emilia-Romagna, Friuli Venezia Giulia, Liguria, Marche, P.A. Bolzano, P.A. Trento, Toscana, Umbria, Veneto—REPORT 2013. Pisa: ETS; 2013.

[14] Nuti S, Fantini MP, Murante AM. Valutare I Percorsi in Sanità. I Percorsi Della Salute Mentale E Il Percorso Oncologico. Il Mulino; 2014.

[15] Tuscany Regional Committee Resolution March 31 2014, n. 272, “Riordino della Rete chirurgica oncologica toscana: primi indirizzi alle Aziende Sanitarie per la costituzione della Rete dei Centri di Senologia e requisiti organizzativo-assistenziali degli stessi”.

[16] Powell AE, Davies HTO, Thomson RG (2003). Using routine comparative data to assess the quality of health care: understanding and avoiding common pitfalls. Qual Saf Health Care.

[17] Flamholtz EG, Das TK, Tsui AS (1985). Toward an integrative framework of organizational control. Account Organ Soc.

[18] Otley D (1999). Performance management: a framework for management control systems research. Manag Account Res.

[19] Krotneva SP, Reidel KE, Verma A, Mayo N, Tamblyn R, Meguerditchian AN (2013). Factors influencing the quality of local management of ductal carcinoma in situ: a cohort study. Curr Oncol.

[20] Peltoniemi P, Peltola M, Hakulinen T, Häkkinen U, Pylkkänen L, Holli K (2011). The effect of hospital volume on the outcome of breast cancer surgery. Ann Surg Oncol.

[21] McDermott AM, Wall DM, Waters PS, Cheung S, Sibbering M, Horgan K, Kearins O, Lawrence G, Patnick J, Kerin MJ (2013). Surgeon and breast unit volume-outcome relationships in breast cancer surgery and treatment. Ann Surg.

[22] Vrijens F, Stordeur S, Beirens K, Devriese S, Van Eycken E, Vlayen J (2012). Effect of hospital volume on processes of care and 5-year survival after breast cancer: a population-based study on 25000 women. Breast.

[23] Nuti S, Seghieri C, Vainieri M (2012). Assessing the effectiveness of a performance evaluation system in the public health care sector: some novel evidence from the Tuscany region experience. J Manag Gov.

[24] Reinertsen JL, Rupp W, Whittington J (2007). Engaging physicians in a shared quality Agenda.

[25] Spurgeon P, Mazelan PM, Barwell F (2011). Medical engagement: a crucial underpinning to organizational performance. Health Serv Manage Res.

[26] Clark J (2012). Medical leadership and engagement: no longer an optional extra. J Health Organ Manag.

[27] Gray M, El Turabi A (2012). Optimising the value of interventions for populations. BMJ.

[28] Wilkinson J, Powell A (2011). Are clinicians engaged in quality improvement ?.

[29] Coopey SB, Tang R, Lei L, Freer PE, Kansal K, Colwell AS, Gadd MA, Specht MC, Austen WG, Smith BL (2013). Increasing eligibility for nipple-sparing mastectomy. Ann Surg Oncol.

[30] Garcia-Etienne CA, Forcellini D, Sagona A, Caviggioli F, Barbieri E, Cornegliani G, Giannasi S, Tinterri C (2012). Breast reconstruction: a quality measure for breast cancer care?. Breast.

[31] Yang RL, Newman AS, Lin IC, Reinke CE, Karakousis GC, Czerniecki BJ, Wu LC, Kelz RR (2013). Trends in immediate breast reconstruction across insurance groups after enactment of breast cancer legislation. Cancer.

[32] Yuen E, Louis D, Cisbani L, Rabinowitz C, De Palma R, Maio V, Leoni M, Grilli R (2011). Using administrative data to identify and stage breast cancer cases: implications for assessing quality of care. Tumori.

[33] Bevan G, Hood C (2006). What’s measured is what matters: targets and gaming in the English public health care system. Public Adm.

[34] D. lgs. July 30 2003, n. 196, “Codice in materia di protezione dei dati personali”.

